# 2-(Thio­phen-2-yl)-*N*-(4-{(*E*)-[2-(thio­phen-2-yl)eth­yl]imino­meth­yl}benzyl­idene)ethanamine

**DOI:** 10.1107/S1600536811009810

**Published:** 2011-03-19

**Authors:** Haleden Chiririwa, Reinout Meijboom, Bernard Omondi

**Affiliations:** a Department of Chemistry, University of Cape Town, Private Bag, Rondebosch 7707, South Africa; b Research Centre for Synthesis and Catalysis, Department of Chemistry, University of Johannesburg, PO Box 524 Auckland Park, Johannesburg 2006, South Africa

## Abstract

In the crystal of the centrosymmetric title compound, C_20_H_20_N_2_S_2_, mol­ecules are linked by head-to-tail C—H⋯N hydrogen bonds, resulting in chains extending along the *a* axis. Three additional C—H⋯π inter­molecular inter­actions give rise to a herringbone packing motif which extends along the *c* axis. The C—H⋯N inter­actions provide links between the sheets.

## Related literature

For related literature on bidendate Schiff base ligands, see: Chakraborty *et al.* (1999 ▶); Haga & Koizumi (1985 ▶).
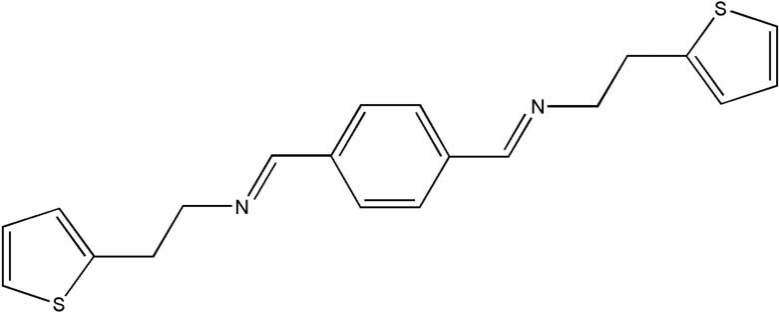


## Experimental

### 

#### Crystal data

C_20_H_20_N_2_S_2_
*M* *_r_* = 352.52
Monoclinic, 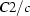

*a* = 9.8592 (10) Å
*b* = 7.1533 (6) Å
*c* = 25.678 (2) Å
β = 96.646 (5)°
*V* = 1798.8 (3) Å^3^
*Z* = 4
Mo *K*α radiation
μ = 0.30 mm^−1^
*T* = 173 K
0.22 × 0.2 × 0.04 mm

#### Data collection

Nonius Kappa CCD diffractometer
Absorption correction: multi-scan (*SADABS*; Bruker, 2007 ▶) *T*_min_ = 0.925, *T*_max_ = 0.988
16248 measured reflections
2230 independent reflections
1679 reflections with *I* > 2σ(*I*)
*R*_int_ = 0.045

#### Refinement

*R*[*F* ^2^ > 2σ(*F* ^2^)] = 0.057
*wR*(*F* ^2^) = 0.181
*S* = 1.08
2230 reflections
109 parameters
14 restraints
H-atom parameters constrained
Δρ_max_ = 0.80 e Å^−3^
Δρ_min_ = −0.42 e Å^−3^

### 

Data collection: *COLLECT* (Nonius, 1998 ▶); cell refinement: *DENZO-SMN* (Otwinowski & Minor, 1997 ▶); data reduction: *DENZO-SMN*; program(s) used to solve structure: *SHELXS97* (Sheldrick, 2008 ▶); program(s) used to refine structure: *SHELXL97* (Sheldrick, 2008 ▶); molecular graphics: *DIAMOND* (Brandenburg & Putz, 2005 ▶) and *ORTEP-3* (Farrugia, 1997 ▶); software used to prepare material for publication: *WinGX* (Farrugia, 1999 ▶).

## Supplementary Material

Crystal structure: contains datablocks global, I. DOI: 10.1107/S1600536811009810/go2007sup1.cif

Structure factors: contains datablocks I. DOI: 10.1107/S1600536811009810/go2007Isup2.hkl

Additional supplementary materials:  crystallographic information; 3D view; checkCIF report

## Figures and Tables

**Table 1:** Hydrogen-bond geometry (Å, °) *Cg*1 andCg2 are the centroids of the thio­phene and benzene rings, respectively.

*D*—H⋯*A*	*D*—H	H⋯*A*	*D*⋯*A*	*D*—H⋯*A*
C4—H4⋯N8^i^	0.95	2.61	3.514 (3)	159
C2—H2⋯*Cg*1^ii^	0.95	2.79	3.702 (3)	161
C6—H6*A*⋯*Cg*2^iii^	0.99	2.72	3.515 (3)	137
C6—H6*A*⋯*Cg*2^iv^	0.99	2.72	3.515 (3)	137

Symmetry codes: (i) 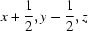; (ii) 
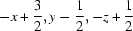
; (iii) 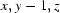; (iv) 
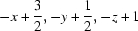
.

**Fig. 1.**
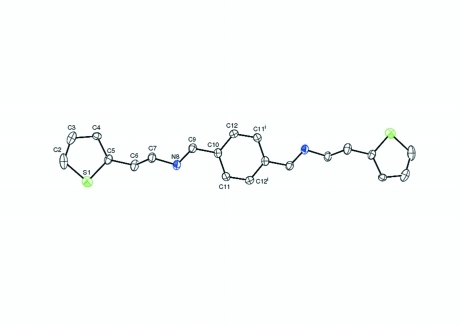
View of (I) (50% probability displacement ellipsoids) with H atoms presented as small spheres of arbitrary radii.

**Fig. 2.**
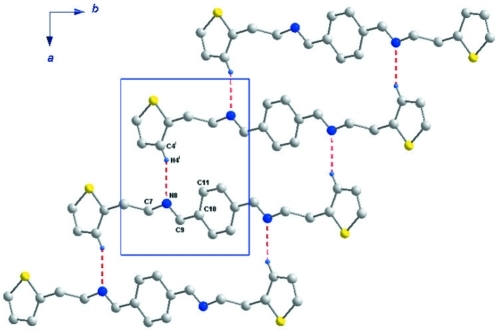
C—H···N hydrogen bond interactions in the crystal structure of (I). [Symmetry operators: (i) = -1/2 + x, 1/2 + y, z]

**Fig. 3.**
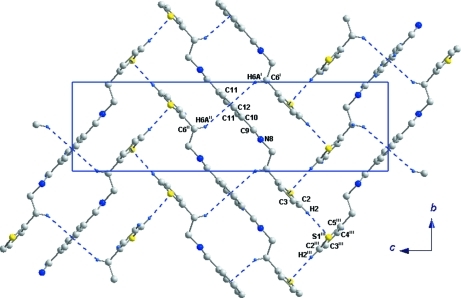
Sheets of C—H···π intermolecular interactions between molecules alligned along the bc face.

## supplementary crystallographic information

### Comment

The title compound belongs to a class of tetradentate ligands. To the best of our knowledge, this is the first example of a neutral thiophenyldimine-based bridging ligand. This compound is a potential tetra-coordinate ligand but on complexation the compound will probably behave as a bidentate ligand as the sulfur, on the thiophene, has weak donor capacity towards co-ordination for majority of metal ions. Besides its use as a ligand, it is interesting from the crystal engineering point of view for the analysis of the packing mode of (I).

Compound (I) crystallizes with half a molecule in the asymmetric unit, with the other half generated through symmetry located in the center of the phenyl ring (Fig. 1). The phenyl ring together with the atoms C7—N8—C9 and the thiophene ring together with the atom C6 are planar with N8 and C5 deviating the most from the planes by 0.018 (2) Å and 0.010 (2) Å respectively. The two planes are close to parallel, the angle between them being 9.3 (1)°. Bond distances and angles in (I) are as expected from the chemical bonding.

The crystal structure of (I) is composed of head-to-tail C—H···N hydrogen bonded chains (Table 1) that extend in the crystallographic *a* axis (Fig. 2). Additionally, the phenyl and thiophen rings are involved in C—H···π intermolecular interactions that result in a herringbone motif that spreads along the crystallographic *c* axis (Fig. 3). The C—H···N interactions are found to connect these herringbone sheets along the *a* axis.,

### Experimental

A solution of benzene 1,4-dicarboxaldehyde (0.50 g, 3.73 mmol) in methanol (10 ml) was added dropwise to a stirred solution of 2-thiophenylethylamine (0.95 g, 7.42 mmol) in methanol (10 ml). The mixture was stirred at room temperature for *ca* 16 h. The precipitate was filtered off and washed with diethylether and dried under vacuum for 4 h affording a fine shiny white powder in 80% yield. *M*.p.: 240–242 °C. Recrystallization was done by slow diffusion of Et_2_O into a concentrated CH_2_Cl_2_ solution of the white powder to give colorless crystals fo (I).

### Refinement

The methine and aromatic H atoms were placed in geometrically idealized positions and constrained to ride on their parent atoms, with C—H = 0.95 Å and *U*_iso_(H) = 1.2*U*_eq_(C) for aromatic, C—H = 0.99 Å and *U*_iso_(H) = 1.2*U*_eq_(C) for CH_2_ C—H = 0.95 Å and *U*_iso_(H) = 1.2*U*_eq_(C) for CH.

### Crystal data

**Table:**

C_20_H_20_N_2_S_2_	*F*(000) = 744
*M**_r_* = 352.52	*D*_x_ = 1.302 Mg m^−^^3^
Monoclinic, *C*2/*c*	Mo *K*α radiation, λ = 0.71073 Å
Hall symbol: -C 2yc	Cell parameters from 34223 reflections
*a* = 9.8592 (10) Å	θ = 3.2–28.3°
*b* = 7.1533 (6) Å	µ = 0.30 mm^−^^1^
*c* = 25.678 (2) Å	*T* = 173 K
β = 96.646 (5)°	Plate, colourless
*V* = 1798.8 (3) Å^3^	0.22 × 0.2 × 0.04 mm
*Z* = 4	

### Data collection

**Table:**

Nonius Kappa CCD diffractometer	*R*_int_ = 0.045
graphite	θ_max_ = 28.3°, θ_min_ = 3.2°
1.0° ω scans, 60s	*h* = −13→13
16248 measured reflections	*k* = −9→9
2230 independent reflections	*l* = −34→34
1679 reflections with *I* > 2σ(*I*)	

### Refinement

**Table:**

Refinement on *F*^2^	Primary atom site location: structure-invariant direct methods
Least-squares matrix: full	Secondary atom site location: difference Fourier map
*R*[*F*^2^ > 2σ(*F*^2^)] = 0.057	Hydrogen site location: inferred from neighbouring sites
*wR*(*F*^2^) = 0.181	H-atom parameters constrained
*S* = 1.08	*w* = 1/[σ^2^(*F*_o_^2^) + (0.0971*P*)^2^ + 3.1807*P*] where *P* = (*F*_o_^2^ + 2*F*_c_^2^)/3
2230 reflections	(Δ/σ)_max_ < 0.001
109 parameters	Δρ_max_ = 0.80 e Å^−^^3^
14 restraints	Δρ_min_ = −0.42 e Å^−^^3^

### Special details

**Table:**

Experimental. The intensity data was collected on a Nonius Kappa CCD diffractometer using an exposure time of 60 sec/per frame. Analytical data: IR (KBr): 1613?cm-1 (C=N, imine); 1H NMR: (CDCl3) δ H 8.23 (d, 2H) 7.76 (s, 2H) 7.13 (dd, 2H) 6.92 (dd, 2H) 6.84 (dd, 4H) 3.91 (dt, 4H) 3.25 (t, 4H); Anal. calcd. for C20H20N2S2: C, 68.14%; H, 5.72%; N, 7.95%; S, 18.19; Found: C, 68.19%; H, 5.52%; N, 7.72%; S, 18.44; EI—MS: m/z 351.76 [M]+;
Geometry. All e.s.d.'s (except the e.s.d. in the dihedral angle between two l.s. planes) are estimated using the full covariance matrix. The cell e.s.d.'s are taken into account individually in the estimation of e.s.d.'s in distances, angles and torsion angles; correlations between e.s.d.'s in cell parameters are only used when they are defined by crystal symmetry. An approximate (isotropic) treatment of cell e.s.d.'s is used for estimating e.s.d.'s involving l.s. planes.
Refinement. Refinement of *F*^2^ against ALL reflections. The weighted *R*-factor *wR* and goodness of fit *S* are based on *F*^2^, conventional *R*-factors *R* are based on *F*, with *F* set to zero for negative *F*^2^. The threshold expression of *F*^2^ > σ(*F*^2^) is used only for calculating *R*-factors(gt) *etc*. and is not relevant to the choice of reflections for refinement. *R*-factors based on *F*^2^ are statistically about twice as large as those based on *F*, and *R*- factors based on ALL data will be even larger.

### Fractional atomic coordinates and isotropic or equivalent isotropic displacement parameters (Å^2^)

**Table:**

	*x*	*y*	*z*	*U*_iso_*/*U*_eq_	
S1	0.62222 (7)	−0.24504 (10)	0.31079 (3)	0.0357 (3)	
C2	0.7356 (3)	−0.3763 (4)	0.28220 (10)	0.0400 (7)	
H2	0.711	−0.4684	0.2562	0.048*	
C3	0.8651 (3)	−0.3341 (4)	0.30095 (10)	0.0355 (6)	
H3	0.9419	−0.3955	0.2896	0.043*	
C4	0.8766 (2)	−0.1915 (3)	0.33866 (8)	0.0194 (4)	
H4	0.9607	−0.1441	0.3552	0.023*	
C5	0.7453 (2)	−0.1270 (3)	0.34886 (9)	0.0230 (5)	
C6	0.7117 (3)	0.0246 (3)	0.38573 (10)	0.0286 (5)	
H6A	0.7616	0.0005	0.4208	0.034*	
H6B	0.6128	0.0204	0.3891	0.034*	
C7	0.7483 (3)	0.2186 (3)	0.36770 (10)	0.0266 (5)	
H7A	0.8479	0.2266	0.366	0.032*	
H7B	0.701	0.2431	0.3322	0.032*	
N8	0.7077 (2)	0.3574 (3)	0.40432 (8)	0.0257 (5)	
C9	0.8002 (2)	0.4632 (3)	0.42630 (9)	0.0232 (5)	
H9	0.891	0.4474	0.4181	0.028*	
C10	0.7729 (2)	0.6096 (3)	0.46404 (9)	0.0220 (5)	
C11	0.6408 (2)	0.6450 (3)	0.47609 (9)	0.0235 (5)	
H11	0.5661	0.5741	0.4598	0.028*	
C12	0.8813 (2)	0.7162 (3)	0.48807 (9)	0.0234 (5)	
H12	0.9713	0.6935	0.4798	0.028*	

### Atomic displacement parameters (Å^2^)

**Table:**

	*U*^11^	*U*^22^	*U*^33^	*U*^12^	*U*^13^	*U*^23^
S1	0.0339 (4)	0.0352 (4)	0.0373 (4)	−0.0022 (3)	0.0007 (3)	−0.0044 (3)
C2	0.073 (2)	0.0227 (13)	0.0245 (12)	−0.0002 (13)	0.0045 (13)	−0.0058 (10)
C3	0.0487 (16)	0.0284 (13)	0.0314 (13)	0.0127 (12)	0.0133 (12)	−0.0009 (9)
C4	0.0167 (9)	0.0190 (10)	0.0217 (10)	0.0007 (8)	−0.0012 (8)	0.0046 (7)
C5	0.0288 (11)	0.0183 (11)	0.0230 (11)	0.0037 (9)	0.0073 (9)	0.0019 (9)
C6	0.0396 (14)	0.0218 (12)	0.0262 (12)	0.0020 (10)	0.0118 (10)	−0.0013 (9)
C7	0.0309 (13)	0.0223 (12)	0.0282 (12)	−0.0010 (9)	0.0096 (10)	−0.0063 (9)
N8	0.0293 (11)	0.0214 (10)	0.0269 (10)	0.0010 (8)	0.0055 (8)	−0.0061 (8)
C9	0.0260 (11)	0.0207 (11)	0.0238 (11)	0.0007 (9)	0.0073 (9)	−0.0010 (9)
C10	0.0269 (12)	0.0181 (11)	0.0211 (10)	−0.0002 (9)	0.0028 (8)	−0.0003 (9)
C11	0.0236 (11)	0.0220 (11)	0.0248 (11)	−0.0024 (9)	0.0026 (9)	−0.0030 (9)
C12	0.0200 (11)	0.0245 (12)	0.0263 (11)	0.0009 (9)	0.0048 (9)	−0.0015 (9)

### Geometric parameters (Å, °)

**Table:**

S1—C2	1.691 (3)	C7—N8	1.455 (3)
S1—C5	1.693 (2)	C7—H7A	0.99
C2—C3	1.345 (4)	C7—H7B	0.99
C2—H2	0.95	N8—C9	1.266 (3)
C3—C4	1.402 (4)	C9—C10	1.472 (3)
C3—H3	0.95	C9—H9	0.95
C4—C5	1.427 (3)	C10—C11	1.397 (3)
C4—H4	0.95	C10—C12	1.397 (3)
C5—C6	1.501 (3)	C11—C12^i^	1.388 (3)
C6—C7	1.520 (3)	C11—H11	0.95
C6—H6A	0.99	C12—C11^i^	1.388 (3)
C6—H6B	0.99	C12—H12	0.95
			
C2—S1—C5	93.55 (13)	N8—C7—C6	109.48 (19)
C3—C2—S1	111.6 (2)	N8—C7—H7A	109.8
C3—C2—H2	124.2	C6—C7—H7A	109.8
S1—C2—H2	124.2	N8—C7—H7B	109.8
C2—C3—C4	114.1 (2)	C6—C7—H7B	109.8
C2—C3—H3	122.9	H7A—C7—H7B	108.2
C4—C3—H3	122.9	C9—N8—C7	117.3 (2)
C3—C4—C5	111.0 (2)	N8—C9—C10	122.8 (2)
C3—C4—H4	124.5	N8—C9—H9	118.6
C5—C4—H4	124.5	C10—C9—H9	118.6
C4—C5—C6	128.3 (2)	C11—C10—C12	119.2 (2)
C4—C5—S1	109.73 (17)	C11—C10—C9	121.4 (2)
C6—C5—S1	121.93 (18)	C12—C10—C9	119.4 (2)
C5—C6—C7	113.0 (2)	C12^i^—C11—C10	119.9 (2)
C5—C6—H6A	109	C12^i^—C11—H11	120
C7—C6—H6A	109	C10—C11—H11	120
C5—C6—H6B	109	C11^i^—C12—C10	120.8 (2)
C7—C6—H6B	109	C11^i^—C12—H12	119.6
H6A—C6—H6B	107.8	C10—C12—H12	119.6
			
C5—S1—C2—C3	0.4 (2)	C5—C6—C7—N8	177.7 (2)
S1—C2—C3—C4	−0.9 (3)	C6—C7—N8—C9	121.8 (2)
C2—C3—C4—C5	1.1 (3)	C7—N8—C9—C10	179.9 (2)
C3—C4—C5—C6	−179.2 (2)	N8—C9—C10—C11	−2.7 (4)
C3—C4—C5—S1	−0.8 (2)	N8—C9—C10—C12	177.7 (2)
C2—S1—C5—C4	0.25 (18)	C12—C10—C11—C12^i^	−0.5 (4)
C2—S1—C5—C6	178.7 (2)	C9—C10—C11—C12^i^	179.9 (2)
C4—C5—C6—C7	69.3 (3)	C11—C10—C12—C11^i^	0.6 (4)
S1—C5—C6—C7	−108.9 (2)	C9—C10—C12—C11^i^	−179.9 (2)

Symmetry codes: (i) −*x*+3/2, −*y*+3/2, −*z*+1.

### Hydrogen-bond geometry (Å, °)

**Table:**

Cg1 andCg2 are the centroids of the thiophene and benzene rings, respectively.

**Table:**

*D*—H···*A*	*D*—H	H···*A*	*D*···*A*	*D*—H···*A*
C4—H4···N8^ii^	0.95	2.61	3.514 (3)	159
C2—H2···Cg1^iii^	0.95	2.79	3.702 (3)	161
C6—H6A···Cg2^iv^	0.99	2.72	3.515 (3)	137
C6—H6A···Cg2^v^	0.99	2.72	3.515 (3)	137

Symmetry codes: (ii) *x*+1/2, *y*−1/2, *z*; (iii) −*x*+3/2, *y*−1/2, −*z*+1/2; (iv) *x*, *y*−1, *z*; (v) −*x*+3/2, −*y*+1/2, −*z*+1.
